# Clinical and Instrumental Characteristics of Newly Diagnosed Patients with Various Forms of Pulmonary Hypertension according to the Russian National Registry

**DOI:** 10.1155/2020/6836973

**Published:** 2020-06-14

**Authors:** Irina Yev. Chazova, Tamila V. Martynyuk, Zarina S. Valieva, Svetlana Yev. Gratsianskaya, Anna M. Aleevskaya, Andrey V. Zorin, Sergey N. Nakonechnikov

**Affiliations:** ^1^Federal State Budgetary Institution, National Medical Research Center of Cardiology of the Ministry of Healthcare of Russian Federation, Russia; ^2^Scientific Research Institute of Clinical Cardiology named after A.L. Myasnikov, Russia; ^3^Department of Pulmonary Hypertension and Heart Diseases, Moscow, Russia; ^4^Faculty of Additional Professional Education, Russian National Research Medical University named after N.I. Pirogov, Russia

## Abstract

**Aim:**

To study demographic and clinical characteristics and to give a comparative description of the functional and hemodynamic status, profile of concomitant pathology in patients with various forms of pulmonary arterial hypertension (PAH), and chronic thromboembolic pulmonary hypertension (CTEPH) according to the Russian National Registry.

**Methods:**

During the period from January 01, 2012, till January 01, 2019, 1105 patients aged >18 years with verified diagnosis of PAH and CTEPH, who were subsequently observed at 15 PH expert centers of the Russian Federation in the 52 provinces, are included in the Russian registry on the basis of the Federal State Budgetary Institution of Cardiology of the Ministry of Healthcare of Russia. All newly diagnosed patients (*n* = 727) were entered into the registry database (NCT03707561). A comparative analysis of demographic and clinical characteristics, profile of concomitant pathology, and parameters of a comprehensive examination of patients was performed.

**Results:**

Among newly diagnosed patients, 67% had PAH and 28.3% had CTEPH. In the PAH group, 40.9% of patients had idiopathic arterial PAH (IPAH), 36.6% had PAH associated with simple congenital heart disease (PAH-CHD), 19.3% had PAH associated with systemic connective tissue disease (PAH-CTD), 1.8% had portal pulmonary hypertension (PoPH), 0.6% had PAH associated with HIV infection (PAH-HIV), 0.4% had heritable PAH (HPAH), and 0.4% had drug/toxin-induced PAH. At the time of diagnosis, PAH patients were younger than patients with CTEPH (45.2 ± 14.9; 52.6 ± 15.3 years, respectively) (*p* < 0.05). At the time of diagnosis, 71% PAH and 77% CTEPH patients had WHO FC III/IV. Mean (±SD) 6MWD was significantly less in CTEPH vs. the PAH group 331.3 ± 110.3 vs. 361.8 ± 135.7 m (*p* = 0.0006). Echo data showed a comparable sPAP between groups; CTEPH population had a more pronounced increase in the area of the right atrium (S_RA_) (24 [20; 32] cm^2^ and 19 [15; 26] cm^2^, respectively), and a significant decrease in FAC (24.7 [22, 4; 29.0] and 29.0 [23.0; 31.0] %, respectively) as compared to the PAH group. RHC showed a comparable increase of sPAP and mPAP in PAH and CTEPH groups. 15.2% of patients with IPAH and HPAH demonstrated positive results in the acute vasoreactivity testing. CTEPH patients were more often obese and suffered from arterial hypertension and right heart failure. Deep venous thrombosis was significantly more often observed in patients with CTEPH (53%). The most common concomitant pathology was erosive-ulcerative lesion of the stomach/duodenum, less often of the esophagus (23.5% and 44.5%, respectively).

**Conclusion:**

According to the Russian registry in patients with PAH and IPAH, the diagnosis is established at a younger age in comparison with the European registries. CTEPH patients are characterized by more severe functional status, pronounced signs of right heart failure taking into account the older age and the spectrum of comorbid pathology, which limits the possibility of surgical treatment. An increase in the number of expert centers participating in the registry is the key to improving early diagnosis of PH and optimal follow-up according to common standards in order to timely optimize therapy and reduce mortality of patients.

## 1. Introduction

Among various forms of pulmonary hypertension, which is determined by an increase of pulmonary artery pressure (PAP), pulmonary arterial hypertension (PAH) and chronic thromboembolic pulmonary hypertension (CTEPH) represent forms of special interest. Both specified forms correspond to the hemodynamic type of precapillary pulmonary hypertension (PH), which is characterized by an increase of mean PAP (mPAP) ≥ 25 mmHg and pulmonary artery wedge pressure (PAWP) ≤ 15 mmHg when measured at rest, by right heart catheterization (RHC) [1, 2].

PAH (group I of clinical classification) is diagnosed when these parameters of central hemodynamics are detected in combination with an increase of pulmonary vascular resistance (PVR) ≥ 3 Wood units (WU) in the absence of other causes of PH, such as lung diseases (group III), previous pulmonary embolism (PE) (group IV), and other rare pathologies (group V) [2, 3]. Group I includes idiopathic (IPAH) and heritable forms of PAH (HPAH), PAH induced by drugs and toxins, and a number of associated subtypes, such as PAH associated with systemic connective tissue disease (PAH-CTD), congenital heart disease (PAH-CHD), portal hypertension (PoPH), HIV infection (PAH-HIV), and schistosomiasis [1, 4].

СTEPH is a distinct form of PH (group IV of clinical classification), in which an increase of PVR and PAP is usually considered as a consequence of stenosis/occlusion of pulmonary arteries by organized thrombotic masses [5, 6]. This is a special, potentially curable by pulmonary thrombendarterectomy (PTE) form of pathology. Notably, the surgical PTE is possible in approximately 60% of all CTEPH patients according to the European Registry [7]. The diagnosis of CTEPH is established in the presence of hemodynamic criteria for precapillary PH and at least three months after the start of effective anticoagulant therapy. For the diagnosis verification, it is also important to detect at least one segmental perfusion defect, according to ventilation-perfusion lung scan, or pulmonary artery obstruction, according to pulmonary CT-angiography [5].

To study the PH patients' epidemiological data and their long-term prognosis, to improve diagnostic and therapeutic approaches irrespective of PH etiology, patient registries are being created across the globe [8–11]. In a number of them, both PAH and CTEPH patients are eligible to registry inclusion, as, for example, in the widely known Portuguese, Swedish, or Spanish registries [11–13].

In 2012, on the basis of the Federal State Budgetary Organization Scientific Research Center of Cardiology of the Ministry of Healthcare of Russia, a national electronic registry has been created, in which data on patients with PAH and CTEPH are entered. In 2017, in order to expand research capabilities, the registry was modernized with the transition to a new platform (https://www.medibase.pro) with a higher productivity and speed while maintaining key functional advantages such as remote access for many users without a need of software installation, entry of unlimited number of patients, data export, timely backup, data protection, etc. [14, 15].

Early diagnosis of PH and differential diagnostic approach in certified PH expert centers make it possible to choose the right treatment strategy, which in turn plays a key role in improving clinical outcomes. It is known that the clinical symptoms of PAH and CTEPH are nonspecific. This factor complicates timely diagnosis and start of targeted therapy. Often, the disease progresses significantly by the time the diagnosis is established, and the treatment starts. Severe significant signs and symptoms of right ventricular heart failure could be observed [9, 16].

Therefore, the study objective was to define demographic and clinical characteristics, to give a comparative description of the functional and hemodynamic status, profile of concomitant pathology, and instrumental and laboratory examination data of patients with various forms of PAH and CTEPH according to the Russian National Registry of Pulmonary Hypertension Patients (https://www.clinicaltrials.gov number NCT03707561).

## 2. Methods

From January 01, 2012, till January 01, 2019, the Russian National Registry included 1105 patients with a newly verified diagnosis of PH and prevalent forms. All patients were hospitalized in the Clinical Cardiology Research Institute named after A.L. Myasnikov, Federal State Budgetary Institution “National Medical Research Center of Cardiology” of the Ministry of Healthcare of Russia. The diagnosis was established by European (2009, 2015) and Russian (2013, 2016) clinical guidelines for the diagnosis and management of PH [1, 2, 5]. Registration of patients who signed an informed consent for participation and processing of personal data was carried out by employees of the Department of Pulmonary Hypertension and Heart Diseases of the Clinical Cardiology Institute named after A.L. Myasnikov. Subsequently, follow-up visits were done in a province where particular patient lives in, and data were entered into the database by employees of 15 regional PH expert centers. Registry access was provided via the Internet at https://www.medibase.pro using an individual username and password. Each HCP from selected centers was processing the following data, i.e., examination results, assessment of functional ability, symptoms, medical history, medical therapy, etc. Quality control of entered data was carried out by employees of the Clinical Cardiology Research Institute.

The prospective study included newly diagnosed patients over the age of 18 years with PAH and CTEPH. Demographic data, region of residence, complaints at the manifestation of PH and by the time of diagnosis verification, the duration of the period from the onset of symptoms to the diagnosis verification, and associated pathology were specified. Clinical data (symptoms, physical examination), functional status (distance in the 6-minute walk test (6MWD), dyspnea index according to the Borg scale, functional class according to the WHO classification (WHO FC), and hemodynamic parameters measured by RHC were evaluated. The acute pharmacological testing, using inhaled nitric oxide (32%) or iloprost (68%), was mandatory in all patients with IPAH and HPAH at RHC. Chest radiography was used to identify quantitative indices like right root diameter, Moore's and Lupi's coefficients, and cardiothoracic ratio. Parameters of transthoracic echocardiography (Echo), spirometry, and ventilation-perfusion lung scan were registered in all patients. Additionally, multislice pulmonary CT-angiography needed for operability assessment was conducted in CTEPH patients. The patients' operability assessment was performed by multidisciplinary expert team including cardiologist, a cardiac surgeon, an endovascular surgeon, and a pulmonologist. Laboratory examination included complete blood count, blood chemistry test, coagulation test, and D-dimer and NT-proBNP plasma levels. Of the 206 newly diagnosed CTEPH patients, pulmonary endarterectomy (PEA) was performed in 66 (32%) patients; balloon angioplasty sessions of the pulmonary arteries were performed in 15 (7%) patients. The results of the registry were reported using reporting guidelines recommended by the Equator network (http://www.equator-network.org) such as RECORD and STROBE statements, and in accordance with Good Publication Practice principles, third iteration (2015).

*Statistical analysis* of the data was performed using STATISTICA 10.0 (StatSoft, USA). Quantitative variables were described by the number of patients, mean ± SD, and median [25; 75 percentiles]. Qualitative variables were described by absolute and relative frequencies (%). Differences between the groups were considered statistically significant at the value *p* < 0.05. The correspondence of sample distribution curve to normal (Gaussian) distribution was checked by the magnitude of the asymmetry and excess coefficients, and the Kolmogorov-Smirnov criterion. The following methods of statistical analysis were used: *χ*^2^-Pearson criterion (analysis of contingency tables), Student *t*-test, Scheffe test for multiple comparisons (comparison of more than two groups), and nonparametric tests (Wilcoxon test, Mann-Whitney test, and Kruskal-Wallis *H*-test).

## 3. Results

The present study included 727 only newly diagnosed patients at the age of 48.7 ± 16.4 years from 52 provinces of the Russian Federation. 67% of patients were diagnosed with PAH, 28.3% of patients were diagnosed with CTEPH, and only 4.7% had other forms of pathology due to left heart or lung diseases. Of the 487 patients with PAH, 40.9% had idiopathic arterial PAH (IPAH), 36.6% had PAH associated with simple congenital heart defects (PAH-CHD), 19.3% had PAH associated with systemic connective tissue disease (PAH-CTD), 1.8% had portal pulmonary hypertension (PoPH), 0.6% had HIV associated (PAH-HIV), 0.4% had hereditable PAH (HPAH), and 0.4% had drug/toxin-induced PAH. In 2 patients with drug-induced PAH, there was a history of taking interferon *α*: in one case for 6 months for hepatitis C and in the other for 13 months for chronic myeloid leukemia.

Patients with PAH at the time of diagnosis were younger than patients with CTEPH (*p* < 0.05) (Table 1). Patients with PAH-CTD and CTEPH were significantly older as compared to IPAH, PAH-CHD, and PoPH groups. PAH is more common in females (81.3%), while the maximum female : male ratio (8.4 : 1) was determined in PAH-CTD patients.

Median time from symptom onset to the diagnosis verification was the longest in IPAH patients, which significantly exceeded the duration of this period in the PAH-CTD and CTEPH groups (Table 2). The НPAH group was characterized by the shortest period till the diagnosis verification with median 9.9 [5.6; 33.2] months. The mean body mass index (BMI) in CTEPH patients was considered to be higher than that in IPAH and PoPH cohorts.

The frequency of clinical symptoms on the onset of the disease and at the time of diagnosis verification in patients with PAH of different etiology and CTEPH is shown in Table 3. In about half of the cases, patients with IPAH showed a sudden onset of symptoms, while in PAH-CTD, PAH-CHD, and PoPH groups it was observed in 12.8%, 13.5%, and 1.1% of cases, respectively (*p* < 0.05).

When examined PAH patient lips cyanosis or acrocyanosis, clubbed fingers and watch glasses fingers were more often observed vs. the CTEPH group. At the same time, peripheral edema of legs or feet, hepatomegaly, lower extremity varicose veins, and wheezing in the lungs was less common in the PAH group. The frequency of auscultatory sign detection, such as the II tone accent over pulmonary artery, systolic murmur localized at left sternal border, and Graham-Steele murmur, did not differ in patients with PAH and CTEPH. The tricuspid regurgitation and Graham-Steele murmurs were most rarely auscultated in PAH-CTD (74.5%) and PoPH (55.5%) groups (Table 4). Signs of right heart failure (RHF) were significantly less observed in the PAH-CHD group vs. IPAH patients.

When analyzing possible risk factors of PH, it was found that the onset of PAH in a number of patients was noted during pregnancy or after delivery. An association with pregnancy was more often detected in patients with IPAH (11.1%), usually in the 3rd trimester of pregnancy, and in PAH-CHD cohort (9.5%) within 12 months after delivery (Table 5).

At the time of diagnosis, 71% of all PAH patients and 61% in the IPAH group had WHO FC III/IV. By the time of diagnosis verification, 77% of CTEPH patients had WHO FC III or IV, and the mean 6MWD was significantly less than that in the PAH group (*p* = 0.0006) (Table 6). In the CTEPH group, lower SpO_2_ was observed at rest vs. IPAH and PoPH groups. While assessing exercise tolerance, the maximal 6MWD and the lowest Borg index were recorded in patients with PoPH. This group had significantly lower WHO FC vs. PAH-CTD (*p* = 0.042) and IPAH (*p* = 0.04) cohorts.

On a chest X-ray, all patients had signs of PAH (Table 7). The smallest values of the right root diameter (*p* < 0.001) were revealed in PAH-CTD patients vs. IPAH and PAH-CHD groups. The smallest values of Moore's (*p* < 0.05) and Lupi's (*p* < 0.05) coefficients were also observed in the PAH-CTD group vs. the PAH-CHD group. Significant differences when analyzing cardiothoracic indexes were observed between PoPH and PAH-CHD groups (*p* = 0.04).

Echocardiography revealed a more pronounced increase in the right atrium area (SRA) in the CTEPH group as compared with that in the PAH group with a comparable value of respective hemodynamic parameters (Table 8). There was a significant decrease in right ventricular fractional area change (RV FAC) with median 24.7 [22.4; 29.0]% in CTEPH vs. 29.0 [23.0; 31.0]% in PAH cohort. The groups of PAH-CTD and PoPH were characterized by a significantly lower increase in sPAP vs. IPAH and PAH-CHD groups, combined with less right atrium area (S_RA_), right ventricle (RV), the main PA, and its branches. In patients with IPAH in contrast to PAH-CHD and CTEPH groups, pronounced remodeling of the heart with significantly smaller sizes of the left atrium (LA), end-diastolic size of LV was noted. Diastolic index eccentricity of the left ventricle in this group was significantly lower vs. PoPH and PAH-CTD groups.

According to RHC, the increased values of sPAP and mPAP were comparable in PAH and CTEPH groups. sPAP levels measured by echocardiography and RHC showed strong positive correlation (*r* = 0.911; *p* = 0.001). IPAH and PAH-CTD patients were characterized by cardiac output (CO) below normal values, in contrast to PoPH and PAH-CHD. The highest values of cardiac index (СI) were recorded in patients with PAH-CHD and PoPH, whereas the lowest values of PVR were calculated in PoPH and PAH-CTD patients (Table 9). A significant decrease in arterial blood O_2_ saturation was detected in patients with PAH-CHD vs. IPAH, PAH-CTD and PoPH groups. The reduction of venous blood O_2_ saturation in patients with IPAH and CTEPH was comparable and significantly lower than that in the other PAH groups. 15.2% of IPAH and HPAH had positive acute vasoreactivity at RHC.

Hematology assessment showed higher levels of hemoglobin, hematocrit, and red blood cells and lower levels of thrombocytes in PAH patients vs. the CTEPH group (Table 10). According to the blood chemistry test, significantly higher levels of creatinine, urea, potassium, fibrinogen, D-dimer, and C-reactive protein were detected in the PAH group. When analyzing blood biomarkers, a significant increase of NT-proBNP levels was observed in patients with PAH/CTEPH without significant differences between groups.

In assessing the profile of concomitant pathology, it was noted that CTEPH patients were more often obese and had arterial hypertension and RHF at the time of diagnosis (Table 11). Lower extremity deep venous thrombosis (DVT) was significantly more frequently observed in patients with CTEPH (53%). The most common concomitant pathology was erosive-ulcerative lesions of the stomach/duodenum, less often of the esophagus (23.5% and 44.5%, respectively).

## 4. Discussion

Over the 7-year period, 1105 patients aged older than 18 years both with a newly verified and prevalent forms were prospectively included in the Russian National Registry. All patients-participants of the registry from 52 provinces of the Russian Federation have been monitored regularly at the main expert center—the Research Institute of Clinical Cardiology named after A.L. Myasnikov—and 15 regional PH expert centers, while initial records were transferred (https://www.medibase.pro). A specific feature of the Russian registry is the inclusion of PH patients with a focus on groups I and IV of clinical classification. The majority of foreign registries exclusively included patients with PAH or CTEPH [9, 10, 17, 18]. Mixed cohorts of PH patients were described in the COMPERA registry, the Portuguese, Swedish, and Spanish registries [11–13, 19]. By the number of observations, the Russian registry is comparable with the European registries (the French registry, *n* = 674, the UK and Irish registry, *n* = 482) and is the second only to the REVEAL registry (USA, *n* = 3515), which included patients with PAH of various etiologies [10, 17, 18].

The objective of this analysis was to study demographic and clinical characteristics, functional and hemodynamic status, profile of concomitant pathology, and examination data in newly diagnosed patients with various forms of PAH and CTEPH according to the Russian National Registry. As many as 727 patients with a verified diagnosis of PAH and CTEPH were included into the studied cohort, which allowed to conduct a comparative analysis of the baseline characteristics of PAH and CTEPH groups, as well as in subgroups of patients with various PAH etiologies.

By the time of diagnosis verification, CTEPH patients often reach WHO FC IV with the development of severe RHF and multiorgan lesions, which is a contraindication to a possible PTE in technically operable patients [5, 16]. The similarity of clinical symptoms in patients with PAH and CTEPH often leads to false diagnosis and attempts to prescribe specific therapy without PTE. According to our data, from 206 patients with the newly diagnosed CTEPH, PTE was performed in 66 (32%) patients, which is significantly lower than in Europe (50-60%) [7, 16, 20].

In the Russian registry, the most common subtypes of PAH were IPAH (40.9%), PAH-CHD (36.6%), and PAH-CTD (19.3%). The proportion of patients with associated forms was 1.8% for PoPH, 0.6% for PAH-HIV, 0.4% for HPAH, and 0.4% for drug- or toxin-induced PAH; no one PAH for schistosomiasis was recorded. Our data on the proportion of patients with various forms of PAH are consistent with the results of the Swedish PAH registry, in which IPAH, PAH-CTD, and PAH-CHD account for 92.5% of patients [12].

Over the years of reporting PAH cases, we noted a very stable IPAH cohort of 40-42% in the overall group, which is consistent with the data of the American (46.2%), Swedish (42.9%), French (39%), and Portuguese (37%) registries [10–12, 17]. The exception is the Chinese registry, in which PAH-СHD was noted as the most common form of PAH (43%) [21]. Moreover, the frequency of registration of PAH-CHD approximately corresponded to Russian data (19%). In the Spanish registry, the proportion of groups with these associated forms of PAH was comparable (PAH-CHD: 16%, PAH-CTD: 15%) [13]. In the REVEAL registry (USA), the proportion of patients with PAH-CTD was approximately 50% of all PAH-associated groups (49.9%), and in the French registry was 25%, which exceeded the proportion of patients with PAH-CHD (11%) [10, 17]. Systemic sclerosis (SS), available, is the leading cause of PAH-CTD according to all data [1, 17]. The reasons for the distribution of PH patients in the Russian registry may be an untimely diagnosis of CHD in children, which leads to the development of Eisenmenger syndrome in adults, as well as an underestimation of the frequency of PAH in CTD patients.

When assessing demographic data, the mean age of our newly diagnosed patients with PAH was significantly younger (45.2 ± 14.9 years) vs. the registries in France and the USA (50 ± 15 and 53 ± 14 years, respectively), which may be associated with a higher number of elderly population of PAH-CTD in these registries. These data are comparable to the Portuguese registry, in which patients were also significantly younger than in the French cohort and REVEAL (USA) at the time of diagnosis [11]. Interesting, the mean age of Russian patients in recent years has not significantly changed in comparison with our previous data of 2-year follow-up [4]. The Portuguese authors also emphasized that patients with IPAH had become older vs. the NIH registry (USA) (median was 36 years) [9, 11]. In the late 80s of the XX century the NIH prospective registry showed that 8% of patients at the time of diagnosis of primary PH were younger than 20 years, and 9% of patients were older than 60 years [9]. Over the last years, the age of IPAH patients has significantly increased in Western countries, reaching 65 years or even more [10, 19]. According to the French r,egistry, the mean age of patients with IPAH, PAH-SS and PAH-CHD at the time of diagnosis was 52, 56, and 39 years, respectively [17]. In our study, the mean age of IPAH patients at diagnosis was 41.0 ± 12.8 years.

In the Russian registry, newly diagnosed CTEPH patients were significantly older than the PAH group (52.6 ± 15.3 and 45.2 ± 14.9 years, respectively) except PAH-CTD (51.4 ± 13.5 years). The diagnosis of CTEPH was established at the younger age vs. the data of other authors [11, 12, 25]. According to the international registry of patients with CTEPH (27 PH sites in 16 countries, *n* = 679), the median age at the time of diagnosis verification was 63 years [7, 16]. In 2016, in the German CTEPH registry, 392 newly diagnosed patients were prospectively included at the mean age of 63.5 ± 15.0 years (equal ratio of men and women) [20]. According to our data, the female/male ratio in the CTEPH group was 38.8%/61.2% vs. 18.7%/81.3% in the PAH group. Most registries showed a clear predominance of females among PAH patients. Portuguese authors described the lowest proportion of women (65%) in the PAH group, explaining this fact by the lack of female patients taking anorectics [11].

In IPAH patients, the period from the onset of the symptoms to the diagnosis verification was 24 months, which corresponded to the data of the French and REVEAL registries (2.25 and 2.03 years, respectively) [10, 17]. The shortest period of 9.9 months was observed in patients with HPAH, which might be associated with a family history of PAH. The mean time from the onset of the first symptoms to the diagnosis of CTEPH was 12.8 months. According to the Spanish registry REHAP (31 sites, *n* = 162), this period was 2.7 ± 4.3 years [13].

Regardless of PAH etiology in all patients, the first manifestation of the disease was dyspnea on exertion. Syncope was noted in the IPAH more often than in subjects with PAH-CTD and PoPH (27.6% vs. 2.9%, *p* < 0.05). According to the NIH registry, the most frequent first symptoms of IPAH were dyspnea (60%), fatigue, and weakness (65%). In 13% of patients, the disease began with syncope [9]. With the progression of the disease to the time of diagnosis, the frequency of clinical symptoms in all groups increased.

On physical examination, varicose veins, peripheral edema, and ascites were more often observed in CTEPH patients vs. the PAH group. The analysis of risk factors in PAH patients revealed an association between the onset of the disease and emotional stress (32.4%), pregnancy, and acute respiratory viral infection. According to our data, the history of deep vein thrombosis (DVT) and pulmonary embolism as presented in 70% and 27.2% of patients with CTEPH, respectively, which could be considered as signs, triggers for the development and further progression of the disease. Compared to the PAH group, hereditary thrombophilia, antiphospholipid syndrome, cancer, and splenectomy were significantly more frequent in patients with CTEPH. In approximately 50% of patients with deep vein thrombosis, asymptomatic pulmonary embolism was detected, and these results are consistent with the data of Lang et al. [22]. According to the German registry, a history of venous thromboembolism was observed in 76.3% of CTEPH patients. 38.3% of patients had at least one risk factor, i.e., thrombophilia (8.2%), cancer (5.6%), antiphospholipid syndrome (4.6%), pacemaker (2.6%), and splenectomy (1.5%) [20].

CTEPH patients with increased BMI (24.1 ± 2.6 kg/m^2^) were predominated in contrast to IPAH and PoPH groups. Concomitant diseases such as obesity (24%) and arterial hypertension (63%) were recorded more often than in the PAH group. Obesity is an additional factor contributing to dyspnea severity and limiting exercise tolerance in patients with PH. According to Weatherald et al., PAH patients with increased BMI and obesity at the time diagnosis had lower WHO FC, higher values of RAP and PVR, and lower СI measured by RHC, vs. patient normal BMI [23]. In our study, low WHO FC in CTEPH patients could also be explained by a significantly higher BMI, as well as by the presence of RHF in 68% of cases.

The 6MWD in CTEPH patients was significantly less than in the PAH group with minimal SpO_2_ measured before 6MWTing. The CTEPH group was characterized by the largest proportion of severe WHO FC III-IV patients (77%) at the time of diagnosis. Our data are consistent with the data of the Spanish REHAP registry (77%) [13] and the German and Portuguese registries (74.8% and 71% of patients, respectively [11, 20].

Patients' functional characteristics according to the Russian registry are generally comparable with the results of the Spanish registry (6MWD 363 ± 120 m; WHO FC III/IV 69%, respectively); the proportions of PAH patients with FC III/IV in the REVEAL and the Chinese registries were 56% and 54%, respectively [10, 13, 21].

At Echo, all PAH/CTEPH patients showed signs of cardiac remodeling with dilatation of the right heart, most pronounced in patients with IPAH and PAH-CHD. The mean value of sPAP in the CTEPH group was slightly higher than that in the PAH group, with a more pronounced increase in S_RA_, a comparable degree of RV dilatation, and a significant decrease of FAC. Among the indicators of right heart remodeling, one of the most important prognostic parameters in PAH patients is RAS. In the retrospective analysis, Austin et al. showed that S_RA_ increase of more than 18 cm^2^ accompanied by the increase in right atrium pressure and the presence of pericardial effusion was a predictor of an unfavorable prognosis [24].

Patients with PAH-CHD showed the largest sizes of the right heart, the diameter of the pulmonary artery and its branches, which is probably associated with a longer duration of the disease. This explains the features of the chest X-ray parameters, e.g., Moore's and Lupi's coefficients in this group were significantly greater than that in the PAH-CTD group. The patients with PAH-CTD and PoPH had the smallest values of the diameter of the right root, as well as of the Moore's and Lupi's coefficients, which also corresponded to the Echo parameters. PoPH patients at the time of diagnosis are characterized by lower WHO, which is consistent with less pronounced hemodynamic disturbances and slight remodeling of the heart.

At RHC, a significantly higher mPAP was revealed in IPAH and PAH-CHD groups, whereas the CTEPH group had significantly lower SaO_2_ values, which explains the severe FC of these patients.

The hemodynamic profile of PAH patients is consistent with the NIH and French registries [8, 17]. The IPAH and PAH-CHD groups were characterized by the highest values of sPAP and mPAP, while patients with IPAH showed the lowest CO/CI, and patients with PAH-CHD had almost normal CO/CI vs. the PAH-CTD and CTEPH groups. The PoPH group was characterized by the most preserved hemodynamic status with the lowest values of sPAP/mPAP and PVR, and near-normal CO/CI. The frequency of positive vasoreactivity testing in our patients with IPAH and HPAH of 15.2% quite correspond to the results of the French and the Swiss registries with 10.3% and 20% of vasoreactive patients, accordingly [17, 25].

Levels of NT-proBNP at the time of diagnosis were significantly increased in PAH and CTEPH patients without significant differences between the groups. Hematology assessment in PAH patients showed significantly higher values of hemoglobin, hematocrit, and the number of red blood cells, while the platelet count was significantly higher in the CTEPH group. When analyzing parameters of the chemistry blood test, patients of CTEPH had significantly higher values of potassium, creatinine and urea, C-reactive protein, fibrinogen, and D-dimer. According to the lipid profile, there were no significant differences between PAH and CTEPH patients.

Modern registries are aimed at the formation of a biobank and search for new potential therapy targets. A new PAH registry (The United States Pulmonary Hypertension Scientific Registry (USPHSR)) was recently initiated in the United States [26]. It is planned to screen 499 patients with PAH, pulmonary capillary hemangiomatosis, and pulmonary venoocclusive disease to assess demographic parameters, examination data, and profile of risk factors. In the Russian registry, a detailed study of the genome is planned as well.

## 5. Conclusion

According to the Russian registry, patients with PAH and IPAH are diagnosed at a younger age vs. data from foreign authors. As the number of patients increases, the proportion of severe patients with WHO FC III-IV is markedly raised, which is associated with a long period of development of the disease until a diagnosis is established, and a proper therapy starts. The high prevalence of PAH-CHD in adults indicates the need for timely diagnosis of CHD in childhood for surgical treatment. The data from the Russian registry indirectly indicate the need for additional efforts aimed at improving the diagnosis of CTD and other associated forms of PAH in adults. Patients with CTEPH have a more severe functional status, pronounced signs of heart failure taking into account the older age and spectrum of comorbid pathology, which limits the possibility of surgical treatment.

The created digital platform for registering cases of PAH and CTEPH is an important tool for obtaining high-quality data that can be compared with data from foreign registries. The modernization of the Russian registry allows to increase the volume of archived information, which promotes attracting other expert sites to collect primary information from a larger number of patients. An increase in the number of expert centers participating in the registry is the key to improving early diagnosis of PH and optimal follow-up according to common standards in order to timely optimization of specific therapy and mortality reduction.

## Figures and Tables

**Table 1:** Demographic status of patients with PAH and CTEPH.

Age groups	PAH patients (*n* = 487)	CTEPH patients (*n* = 206)
18-44, *n* (% men/women)	299	47
45-59, *n* (% men/women)	168	99
≥60, *n* (% men/women)	20	60
Age depending on gender (mean ± SD)		
Men	42.5 ± 11.9 (*n* = 91)^#^	50.1 ± 14.2 (*n* = 80)
Women	48.8 ± 20.8 (*n* = 396)^#^	55.7 ± 13.9 (*n* = 126)

^#^ *p* < 0.05 vs. the CTEPH subgroup.

**Table 2:** Demographic characteristics of patients with a newly diagnosed PAH and CTEPH in the Russian registry.

Parameters	Patient groups					
	PAH *n* = 487	IPAH *n* = 199	PAH-CTD *n* = 94	PAH-CHD *n* = 178	Other PAH *n* = 16	CTEPH *n* = 206
Age^∗^, years	45.2 ± 14.9^4^	41.0 ± 12.8^4^	51.4 ± 13.5^1.3^	41.2 ± 13.4^4^	40.7 ± 7.4^4^	52.6 ± 15.3
Gender: women, (*n*, %)	396 (81.3%)^4^	168 (84.4%)^4^	84 (74.7%)^4^	133 (89.4%)^4^	11 (68.7%)	126 (61.2%)
BMI (kg/m^2^)	24.6 ± 8.0	23.9 ± 4.9^4^	24.9 ± 3.6	24.4 ± 4.6	23.9 ± 3.1^4^	28.7 ± 14.6
Time from symptom onset to diagnosis (months)	16.9 [3.8; 34.4]	24.0 [8.4; 45.6]^2.4^	10.9 [4.8; 14.4]*^1^*	14.2 [6.0; 33.8]	11.3 [4.7; 43.8]	12.8 [2.5; 43.2]

Note: ∗: at the time of diagnosis; 1: *p*IPAH‐PAH‐CTD < 0.05; 2: *p*IPAH‐PAH‐CHD < 0.05; 3: *p*PAH‐CTDD‐PAH‐CHD < 0.05; 4: *p* vs. CTEPH group < 0.05.

**Table 3:** Clinical symptoms in patients with PAH and CTEPH.

Clinical symptoms	Patient groups											
	IPAH (*n* = 199)		PAH-CTD (*n* = 94)		PAH-CHD (*n* = 178)		PoPH (*n* = 9)		PAH group (*n* = 487)		CTEPH group (*n* = 206)	
	Onset (*n*, %)	Time of diagnosis verification (*n*, %)	Onset (*n*, %)	Time of diagnosis verification (*n*, %)	Onset (*n*, %)	Time of diagnosis verification (*n*, %)	Onset (*n*, %)	Time of diagnosis verification (*n*, %)	Onset (*n*, %)	Time of diagnosis verification (*n*, %)	Onset (*n*, %)	Time of diagnosis verification (*n*, %)
Dyspnea	171 (85.9%)^2^	197 (98.9%)	89 (94.7%)^4^	92 (97.8%)	173 (97.2%)^4^	178 (100%)	7 (77.8%)	9 (100%)^∗^	425 (87.3%)	483 (99.2%)	189 (91.7%)	202 (98.1%)
Fatigue	66 (33.1%)	129 (64.8%)^4^^∗^	32 (34.0%)	72 (76.6%)^∗^	40 (22.5%)^4^	122(68.5%)^4^^∗^	5 (55.6%)	8 (88.9%)^∗^	242 (49.7%)^∗^	362 (74.3%)^#^	63 (30.6%)	163 (79.1%)^#^
Chest pain	34 (17.1%)	82 (41.2%)^∗^	12 (12.8%)^2^	48 (51.2%)^∗^	39 (21.9%)^4^	90 (50.6%)^4^^∗^	1 (11.1%)	3 (33.3%)^∗^	72 (14.8%)^∗^	212 (43.5%)^#^	70 (33.9%)	91 (44.2%)
Dizziness	45 (22.6%)^4^	90 (45.2%)^4^^∗^	22 (23.4%)^4^	38 (40.4%)^4^^∗^	41 (23.0%)^4^	84 (47.2%)^4^^∗^	1 (11.1%)	2 (22.2%)	75 (15.4%)	188 (38.6%)^∗^	21 (10.2%)	110 (53.4%)^#^
Syncope	51 (25.6%)^4^	55 (27.6%)^2.4^	1 (1.1%)^1^	11 (11.7%)^1.4^	14 (7.9%)^4^	22 (12.4%)^4^	0 (0%)	0 (0%)	75 (15.4%)	125 (25.7%)^∗^	40 (19.4%)	25 (12.1%)
Palpitations	76 (38.2%)	128 (64.3%)^2^^∗^	32 (34.0%)	78 (82.9%)^1^^∗^	41 (23.0%)	80 (44.9%)^4^^∗^	2 (22.2%)	7 (77.8%)^∗^	120 (24.6%)	312 (64.1%)^#^	48 (23.3%)	106 (51.5%)^#^
Cough	34 (17.1%)^4^	80 (40.2%)^∗^^2.4^	10 (10.6%)^4^	46 (48.9%)^3.4^^∗^	24 (13.5%)^4^	60 (33.7%)^∗^	0 (0%)	3 (33.3%)^∗^	57 (11.7%)^∗^	187 (38.4%)^∗^	70 (33.9%)	123 (59.7%)^#^
Hemoptysis	3 (1.5%)	4 (2.0%)	0 (0%)	0 (0%)	11 (6.2%)	39 (21.9%)^3.4^^∗^	0 (0%)	0 (0%)	14 (2.9%)^∗^	28 (5.3%)^∗^	25 (12.1%)	50 (24.2%)
Hoarseness	0 (0%)	8 (4.0%)^4^	0 (0%)	1 (1.1%)	0 (0%)	8 (4.5%)^4^	0 (0%)	0 (0%)	0 (0%)	11 (2.3%)	0 (0%)	2 (0.9%)
Swelling of the legs/feet	30 (15.1%)	62 (31.2%)^∗^	15 (15.9%)	32 (34.0%)^∗^	22 (12.4%)	71 (39.9%)^4^^∗^	1 (11.1%)	2 (22.2%)	60 (12.3%)	182 (37.4%)^∗^^#^	46 (22.3%)	146 (68.4%)^#^

^∗^ *p* vs. baseline; 1: *p* IPAH‐PAH‐CTD < 0.05; 2: *p* IPAH‐PAH‐CHD < 0.05; 3: *p* PAH‐CTD‐PAH‐CHD < 0.05; 4: *p* vs. PoPH group < 0.05.

**Table 4:** Examination data for patients with PAH and CTEPH at the time of diagnosis.

Signs	Patient groups					
	PAH *n* = 487	IPAH *n* = 199	PAH-CTD *n* = 94	PAH-CHD *n* = 178	PoPH *n* = 9	CTEPH *n* = 206
Lips cyanosis, acrocyanosis	192 (39.4%)^4^	62 (31.5%)^2.5^	23 (24.5%)^3^	109 (61.2%)^4.5^	1 (11.1%)	46 (22.3%)
Сlubbed fingers/watch-glass nails	47 (9.7%)^4^	3 (1.5%)^2^	0 (0%)^3^	59 (33.1%)^4.5^	0 (0%)	1 (0.5%)
Accentuated P2	471 (96.7%)	197 (98.9%)^5^	92 (97.9%)^5^	177 (99.4%)^5^	6 (66.7%)	192 (93.2%)^5^
Systolic murmur localized at left sternal border	431 (88.5%)	185 (92.9%)^1.5^	70 (74.5%)^3^	163 (91.5%)^5^	5 (55.5%)	168 (81.5%)^5^
Pulmonary insufficiency	112 (22.9%)	45 (22.6%)^1.5^	10 (10.6%)^3.4.5^	48 (26.9%)^5^	0 (0%)	41 (19.9%)^5^
Wheezing	18 (3.7%)^4^	10 (5.1%)^2.4.5^	5 (5.3%)^3.4.5^	1 (0.6%)^4^	0 (0%)	56 (27.2%)^5^
Varicose veins	8 (1.6%)^4^	2 (1.0%)	1 (1.1%)	5 (2.8%)	0 (0%)	18 (8.7%)^5^
Swelling of the legs/feet	106 (27.8%)^4^	71 (35.7%)^1.2.4.5^	18 (19.1%)^4.5^	43 (24.2%)^4.5^	0 (0%)	142 (68.9%)^5^
Hepatomegaly	38 (7.8%)^4^	20 (10.1%)^2.5^	6 (6.4%)	3 (1.8%)^5^	6 (66.7%)	62 (30.1%)^5^
Ascites	9 (1.8%)	8 (4.0%)^1.2.4^	0 (0%)^4^	0 (0%)^4^	0 (0%)	10 (4.8%)^5^

Note: 1: *p* IPAH‐PAH‐CTD < 0.05; 2: *p* IPAH‐PAH‐CHD < 0.05; 3: *p* PAH‐CTD‐PAH‐CHD < 0.05; 4: *p* vs. the CTEPH group < 0.05; 5: *p* vs. the group of PoPH < 0.05.

**Table 5:** Risk factors in patients with PAH and CTEPH.

Factors	Patient groups					
	PAH *n* = 487	IPAH *n* = 199	PAH-CTD *n* = 94	PAH-CHD *n* = 178	PoPH *n* = 9	CTEPH *n* = 206
Association with pregnancy	43 (8.8%)	22 (11.1%)^1,4,5^	1 (1.1%)	17 (9.5%)^3,4,5^	0 (0%)	6 (2.9%)
Onset connection with acute respiratory infection	43 (8.8%)	28 (14.1%)^1,2^	3 (3.3%)	6 (3.4%)	1 (11.1%)	24 (11.7%)
Onset connection with the past stress	43 (32.4%)^4^	98 (49.2%)^1,2,4^	21 (22.3%)	35 (19.6%)^4^	1 (11.1%)	20 (9.7%)
Onset connection with the pulmonary embolism	0 (0%)^4^	0 (0%)^4^	0 (0%)^4^	0 (0%)^4^	0 (0%)^4^	56 (27.2%)

Note: 1: *p* IPAH‐PAH‐CTD < 0.05; 2: *p* IPAH‐PAH‐CHD < 0.05; 3: *p* PAH‐CTD‐PAH‐CHD < 0.05; 4: *p* vs. CTEPH group < 0.05; 5: *p* vs. PoPH < 0.05.

**Table 6:** Assessment of functional status in patients with PH at the time of diagnosis.

Parameters	Patient groups					
	PAH *n* = 487	IPAH *n* = 199	PAH-CTD *n* = 94	PAH-CHD *n* = 178	PoPH *n* = 9	CTEPH *n* = 206
6MWD, meters	361.8 ± 135.7^4^	373.0 ± 119.8^1,4,5^	353.6 ± 86.3^5^	370.7 ± 86.5^4,5^	451.7 ± 24.1^4^	331.3 ± 110.3
Borg dyspnea index scale, points	3.3 ± 1.2^4^	3.5 ± 1.8^2,5^	3.7 ± 1.7^3,5^	2.8 ± 1.4^4^	2.9 ± 0.9^4^	3.8 ± 2.8
SpО_2_ before 6MWD, (%)	95.0 [91.2; 98.0]	96.5 [94.0; 98.0]^4^	95.3 [93.0; 97.1]	92.3 [89.7; 95.0]^2.3.5^	96.3 [94.8; 98.0]^4^	94.0 [90.0; 97.0]
FC (mean ± SD)	2.57 ± 0.72^4^	2.69 ± 0.64^4.5^	2.81 ± 0.82^5^	2.72 ± 0.55^4^	2.44 ± 0.45^4^	3.25 ± 0.55
WHO FC I/II/III/IV	10%/19%/65%/6%	7%/32%/48%/13%	8%/24%/58%/10%	9%/25%/47%/19%	22%/34%/44%/0%	3%/20%/59%/18%

Note: ∗: at the time of diagnosis; 1: *p* IPAH‐PAH‐CTD < 0.05; 2: *p* IPAH‐PAH‐CHD < 0.05; 3: *p* PAH‐CTD‐PAH‐CHD < 0.05; 4: *p* vs. CTEPH < 0.05; 5: *p* vs. PoPH < 0.05.

**Table 7:** Chest Х-ray in patients with PAH and CTEPH.

Parameters	Patient groups						Upper limit of normal value
	PAH *n* = 487	IPAH *n* = 199	PAH-CTD *n* = 94	PAH-CHD *n* = 178	PoPH *n* = 9	CTEPH *n* = 206	
Diameter of the right PA root (cm)	1.9 [1.6; 2.6]	2.0 [1.7; 2.3]	1.7 [1.6; 1.9]^1.3^	2.1 (1.7; 2.6)	1.8 [1.6; 2.2]	1.8 [1.6; 2.3]	≤1.5
Moore's coefficient (%)	35.9 [32; 41]	36.2 [33; 38]^2.4.5^	35.7 [32; 39]^3^	39.7 [35; 43]^5^	33.4 [31; 35]	34.0 [30.0; 38.0]	≤30
Lupi's coefficient (%)	35.6 [32; 39]	35.1 [33; 38]	34.6 [32; 36]^3^	38.6 [36; 42]^4.5^	34.6 [32; 36]	35.0 [33.0; 37.0]	≤33
Cardiothoracic ratio (%)	51.7 [45; 57]	51.8 [48; 55]	50.5 [48; 53]	53.5 [38; 64]^5^	49.3 [43; 51]	51.0 [48; 57.0]	≤50

Note: ∗: at the time of diagnosis; 1: *p* IPAH‐PAH‐CTD < 0.05; 2: *p* IPAH‐PAH‐CHD < 0.05; 3: *p* PAH‐CTD‐PAH‐CHD < 0.05; 4: *p* vs. CTEPH < 0.05; 5: *p* vs. PoPH < 0.05. Moore's coefficient: percentage of the distance from the farthest point of the PA arc to the vertebral midline to the half chest diameter. Lupi's coefficient: the percentage of the sum of the distances from the midline to the first division of the right and left pulmonary arteries to the diameter of the chest.

**Table 8:** Echocardiography parameters in patients with PAH and CTEPH.

Parameters	Patient groups						Control group
	PAH *n* = 487	IPAH *n* = 199	PAH-CTD *n* = 94	PAH-CHD *n* = 178	PoPH *n* = 9	CTEPH *n* = 206	
sPAP (mmHg)	78.0 [70.0; 104.0]^^^	85 [67; 103]^^1.5^	70 [65; 87]^*^*4^	83 [55; 101]^^5^	75.0 [69; 90]^^4^	84 [71; 101]^^^	20.4 ± 2.0
RAS (cm)	19 [15; 26]^^4^	21 [18; 27]^^1.4.5^	18 [16; 23]^^^	22 [17; 28]^^3.5^	17 [16; 25]^^4^	24 [20; 32]^^^	11.0 [10.0; 13.0]
Front-rear RV size (cm)	3.7 [3.2; 4.3]^^^	4.1 [3.9; 4.5]^^1.5^	3.6 [3.9; 4.1]^^4^	4.2 [3.9; 4.5]^^^	3.7 [3.2; 4.2]^4^	3.9 [3.2; 4.6]	2.9 [2.7; 3.1]
RV AWT (cm)	0.68 ± 0.25^^^	0.82 ± 0.21^^5^	0.40 ± 0.09^1.4^	0.88 ± 0.32^^3.5^	0.51 ± 0.18^^^	0.89 ± 0.30^^^	0.31 ± 0.08
TAPSE (cm)	1.7 [1.5; 2.0]^^^	1.6 [1.4; 1.9]^^5^	1.5 [1.3; 1.8]^^5^	1.8 [1.6; 2.0]^^4^	1.9 [1.7; 2.2]^4^	1.4 [1.3; 1.5]^^^	2.2 [1.9; 2.4]
Fractional area change (RVFAC) (%)	29.0 [23.0; 31.0]^^4^	26.0 [22.0; 31.5]^^5^	26.2 [23.8; 29.0]^^5^	32.0 [28.0; 33.0]^^2.3.4^	35.2 [30.0; 37.0]^^4^	24.7 [22.4; 29.0]^^^	45.0 [44.0; 46.0]
LV diastolic eccentricity index	1.3 [1.0; 1.7]^^^	1.4 [1.2; 1.6]^^1.5^	1.2 [1.0; 1.3]	1.3 [1.1; 1.4]^^5^	1.1 [1.0; 1.4]	1.4 [1.2; 1.6]^^^	1.0 [1.0; 1.0]
LA (cm)	3.4 [3.0; 3.9]^4^	3.3 [2.9; 3.7]^4^	3.5 [3.1; 3.9]	3.7 [3.3; 3.9]^^2.5^	3.2 [3.0; 3.5]^4^	3.7 [3.4; 4.0]^^^	3.4 [3.2; 3.6]
LVEDd (cm)	4.2 [3.8; 4.7]^^^	4.0 [3.4; 4.4]^^1.4.5^	4.3 [3.9; 4.7]	4.4 [4.00; 4.6]^2^	4.3 [3.9; 4.8]	4.3 [3.9; 4.6]^^^	4.8 [4.6; 5.0]
Interventricular septum thickness (cm)	0.9 [0.85; 1.0]	0.9 [0.85; 1.0]	1.0 [0.9; 1.1]	1.0 [0.9; 1.1]	0.9 [0.8; 1.0]	1.0 [0.9; 1.1]	0.9 [0.85; 1.0]
Ao (cm)	3.1 [2.7; 3.4]^4^	3.1 [2.8; 3.3]^4^	3.1 [3.0; 3.2]^4^	3.2 [2.8; 3.5]^^^	3.0 [2.7; 3.1]^4^	3.5 [2.7; 4.9]^^^	2.8 [2.6; 3.0)
Pulmonary trunk diameter (cm)	3.3 [3.0; 3.9]^^^	3.3 [2.9; 3.7]^^1.2^	3.0 [2.9; 3.3]^^^	3.5 [3.3; 4.0]^^4.5^	2.8 [2.6; 3.7]^^^	3.2 [2.9; 3.4]^^^	1.7 [1.8; 2.0)
RPA (cm)	2.2 [1.9; 2.5]^^^	2.1 [1.9; 2.5]^2^	2.0 [1.9; 2.3]^^^	2.3 [2.1; 2.8]^^2.3.5^	2.0 [1.9; 2.3]^^4^	2.2 [2.0; 2.4]^^^	1.1 [1.0; 1.4]
LPA (cm)	2.1 [1.9-2.3]^^^	2.0 [1.8; 2.3]^^^	2.2 [2.0; 2.4]^^^	2.3 [1.9; 2.5]^^2.3.5^	1.9 [1.8; 2.2]^^4^	2.2 [2.0; 2.4]^^^	1.2 [0.9; 1.5]

Note: Ao: aorta; LA: left atrium; LVEDd: left ventricular end diastolic dimension; RV APD: anteroposterior size of the right ventricle; RVAWT: the thickness of the anterior wall of the right ventricle; RPA: right pulmonary artery; LPA: left pulmonary artery; sPAP: systolic pressure in the pulmonary artery; TAPSE: Tricuspid Annular Plane Systolic Excursion. ^: vs. the control group (*p* < 0.05);*p* IPAH‐PAH‐CTD < 05.0; 0: *p* IPAH‐PAH‐CHD <2 *p* PAH‐CTD‐PAH‐CHD <0.05; 3:*p*vs.CTEPH group < 1; 5:*p*vs.PoPH < 0.05.

**Table 9:** Hemodynamic parameters according to right heart catheterization in PAH and CTEPH patients.

Parameters	Patient groups						Upper limit of normal value
	PAH *n* = 487	IPAH *n* = 199	PAH-CTD *n* = 94	PAH-CHD *n* = 178	PoPH *n* = 9	CTEPH *n* = 206	
sPAP (mmHg)	82.5 ± 34.1	89.5 ± 25.9^5^	76.0 ± 7.9^1.4^	95.0 ± 25.9^3.5^	72.5 ± 10.9^4^	89.3 ± 21.3	<30-36
Mean PAP (mmHg)	56.1 ± 20.9^4^	58.8 ± 15.5^4.5^	48.0 ± 11.3^1^	61.5 ± 19.3^3^	48.0 ± 8.9	51.4 ± 12.8	<21
Mean RAP (mmHg)	6.8 ± 4.7	6.3 ± 4.5^5^	6.0 ± 5.5^5^	6.4 ± 5.1^5^	5.0 ± 2.1^4^	6.8 ± 4.9	2-6
PAWP (mmHg)	6.8 ± 3.9	6.1 ± 3.8	8.0 ± 2.5^1.5^	8.0 ± 3.7^2.5^	5.5 ± 1.2	6.5 ± 4.1	6-12
CO (l/min)	3.8 ± 1.2	3.5 ± 1.1^5^	4.5 ± 1.3^1.4^	4.7 ± 1.2^2.4^	4.3 ± 0.9^4^	3.5 ± 0.8	4.0–8.0
CI (l/min/m^2^)	2.1 ± 0.6	2.0 ± 0.6	2.3 ± 0.3^1.4^	2.6 ± 0.5^2.4^	2.4 ± 0.3^4^	1.9 ± 0.5	2.5-4.0
PVR (dyn·s/cm^5^)	1105 ± 677.6	1243.9 ± 583.5^4.5^	688.0 ± 577.9^1^	1300.0 ± 571.5^3^	780.0 ± 579.5^4^	1075.8 ± 477.8	<120
SvO_2_ (%)	58.0 ± 8.4	57.8 ± 9.6^5^	64.0 ± 4.6^1.4^	67.0 ± 9.6^2.4^	63.0 ± 2.6^4^	57.7 ± 7.9	70-80
SaО_2_ (%)	94.4 ± 5.2^4^	94.6 ± 2.2^4^	96.0 ± 2.7^4^	92.0 ± 2.2^2.3.5^	96.5 ± 1.0^4^	91.9 ± 4.5	95-100

Note: sPAP: systolic pressure in the pulmonary artery; mean PAP: mean pressure in the pulmonary artery; mean RAP: mean pressure in the right atrium; PAWP: pulmonary artery wedge pressure; CO: cardiac output; CI: cardiac index; PVR: pulmonary vascular resistance; SvO_2_: mixed venous saturation; SaО_2_: blood oxygen saturation. 1: *p* IPAH‐PAH‐CTD < 0.05; 2: *p* IPAH‐PAH‐CHD < 0.05; 3: *p* PAH‐CTD‐PAH‐CHD < 0.05; 4: *p* vs. CTEPH < 0.05; 0: *p* vs. PoPH < 0.05.

**Table 10:** Laboratory indicators in the PAH and CTEPH groups.

Parameters	PAH group (*n* = 487)	CTEPH group (*n* = 206)	*p*
Potassium (mmol/l)	4.51 ± 0.34	4.83 ± 0.43	0.003
Sodium (mmol/l)	140.51 ± 10.12	141.79 ± 4.65	N/S
Creatinine (*μ*mol/l)	82.12 ± 16.80	92.41 ± 18.81	0.0001
Iron (*μ*mol/l)	20.19 ± 16.41	11.55 ± 7.88	N/S
Uric acid (*μ*mol/l)	378.72 ± 138.51	401.42 ± 143.87	N/S
Urea (mmol/l)	5.78 ± 1.91	7.49 ± 2.89	0.000055
Bilirubin total (*μ*mol/l)	23.08 ± 11.22	22.38 ± 10.39	N/S
Glucose (mmol/l)	5.22 ± 1.45	5.52 ± 1.31	N/S
LDL cholesterol (mmol/l)	2.89 ± 0.90	2.91 ± 1.08	N/S
HDL cholesterol (mmol/l)	1.25 ± 0.36	1.27 ± 0.41	N/S
Cholesterol (mmol/l)	4.68 ± 1.10	4.75 ± 1.28	N/S
C-reactive protein (mg/dl)	0.18 [0.09; 0.48]	0.49 [0.32; 1.88]	0.001
Triglycerides (mmol/l)	1.30 ± 0.72	1.38 ± 0.87	N/S
Hemoglobin (g/dl)	14.87 ± 2.09	14.18 ± 2.33	0.031
Hematocrit (%)	44.46 ± 6.28	41.88 ± 8.68	0.006
Red blood cells (10^12^/l)	5.11 ± 0.80	4.94 ± 0.77	0.011
White blood cells (10^9^/l)	7.36 ± 1.88	7.51 ± 2.20	N/S
Platelets (10^9^/l)	217.75 ± 72.68	255.08 ± 114.98	0.0033
ESR (mm/hour)	9.81 ± 14.59	15.32 ± 21.61	0.012
Fibrinogen (g/l)	3.0 ± 0.55	3.41 ± 0.82	0.0023
D-dimer (*μ*g/ml)	0.2 [0.1; 0.30]	0.5 [0.2; 1.3]	0.0005
NT-proBNP (pg/ml)	1188 [275; 4570]	1750 [915; 3055]	N/S

Note: LDL: low-density lipoproteins; HDL: high-density lipoproteins; ESR: erythrocyte sedimentation rate.

**Table 11:** Analysis of concomitant pathology in patients with PH.

Diseases	PAH	CTEPH		PAH	CTEPH
Erosive and ulcerative lesions of the upper gastrointestinal tract	23.5%^∗^	44.5%	Atrial fibrillation/flutter	5.4%	7.8%
Arterial hypertension	18.9%^∗^	63.8%	Other heart rhythm and conduction disorders	3.3%	7.8%
CHF	37%^∗^	68%	Uterine myoma	2.9%	5.8%
Vein thrombosis	1%^∗^	53%	Liver fibrosis/cirrhosis	2.1%	1.6%
Atherosclerotic lesion of the peripheral arteries	2.9%	1.9%	Urolithiasis/nephrolithiasis	8.7%	9.6%
Coronary heart disease	3%	11%	Bladder pathology	1.7%	1.6%
Carbohydrate tolerance disorder	0.8%	5.8%	COPD	2.1%	7.8%
Diabetes mellitus	2.1%	5.8%	Bronchial asthma	2.1%	3.2%
Varicose veins of legs	8.7%	15.4%	Anaemia	2.9%	2.8%
Spinal osteochondrosis	5.0%	3.2%	Epistaxis	1.2%	
Obesity	10%^∗^	24.5%	Phlebitis	4.1%	11%
Nodular goiter	5.0%	9.6%	Ischemic stroke	1.7%	8%
Autoimmune thyroiditis	5.4%	9.6%	History of cancer	0.3%^∗^	7%
Thrombophilia	2.3%	24.5%	CTD	19%	17%
Hypothyroidism	4.1%	3.2%			
Gallbladder disease	5.4%	3.2%			

^∗^ *p* vs. CTEPH group < 0.05.

## Data Availability

The data used to support the findings of this study are included within the article.
